# Systematic Analysis of Pharmaceutical Preparations of Chondroitin Sulfate Combined with Glucosamine

**DOI:** 10.3390/ph10020038

**Published:** 2017-04-01

**Authors:** Gustavo R.C. Santos, Adriana A. Piquet, Bianca F. Glauser, Ana M.F. Tovar, Mariana S. Pereira, Eduardo Vilanova, Paulo A.S. Mourão

**Affiliations:** Laboratório de Tecido Conjuntivo, Hospital Universitário Clementino Fraga Filho and Instituto de Bioquímica Médica Leopoldo de Meis, Universidade Federal do Rio de Janeiro (UFRJ), P.O. Box 68041, Rio de Janeiro RJ 21941-913, Brazil; fgugarc@hotmail.com (G.R.C.S.); piquet@hucff.ufrj.br (A.A.P.); biancaglauser@gmail.com (B.F.G.); tovara@gmail.com (A.M.F.T.); marianasa@hucff.ufrj.br (M.S.P.); evilanova@hucff.ufrj.br (E.V.)

**Keywords:** glycosaminoglycans, glucosamine sulfate, arthritis, arthroses, nuclear magnetic resonance

## Abstract

Glycosaminoglycans are carbohydrate-based compounds widely employed as nutraceuticals or prescribed drugs. Oral formulations of chondroitin sulfate combined with glucosamine sulfate have been increasingly used to treat the symptoms of osteoarthritis and osteoarthrosis. The chondroitin sulfate of these combinations can be obtained from shark or bovine cartilages and hence presents differences regarding the proportions of 4- and 6-sulfated *N*-acetyl β-d-galactosamine units. Herein, we proposed a systematic protocol to assess pharmaceutical batches of this combination drug. Chemical analyses on the amounts of chondroitin sulfate and glucosamine in the batches were in accordance with those declared by the manufacturers. Anion-exchange chromatography has proven more effective than electrophoresis to determine the type of chondroitin sulfate present in the combinations and to detect the presence of keratan sulfate, a common contaminant found in batches prepared with shark chondroitin sulfate. 1D NMR spectra revealed the presence of non-sulfated instead of sulfated glucosamine in the formulations and thus in disagreement with the claims declared on the label. Moreover, 1D and 2D NMR analyses allowed a precise determination on the chemical structures of the chondroitin sulfate present in the formulations. The set of analytical tools suggested here could be useful as guidelines to improve the quality of this medication.

## 1. Introduction

**Figure 1:**
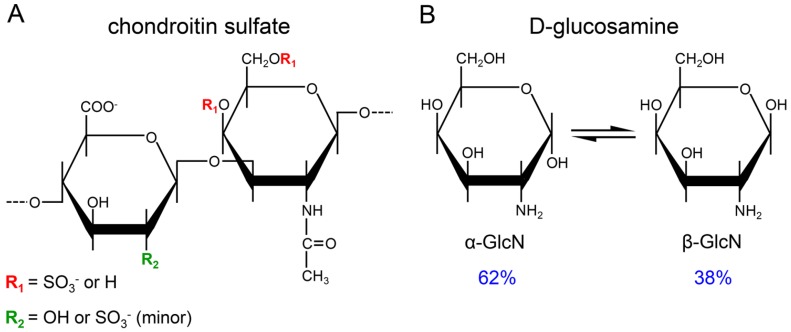
Structure of the active compounds present in the pharmaceutical preparations. (**A**) CS purified from bovine or shark cartilage. The sulfation pattern of the *N*-acetyl β-galactosamine (in red) and β-glucuronic acid (in green) units varies according with the animal source of the CS. Free GlcN (**B**) in solution epimerizes on its α- (62%) and β- (38%) anomers.

Glycosaminoglycans (GAGs) are carbohydrate-based biological compounds widely employed in medicine as therapeutic agents [1]. Currently, heparin stands out as the most exploited GAG drug, being largely used for treatment and prevention of thrombosis and in procedures involving extracorporeal circulation [2]. In recent years, oral formulations of chondroitin sulfate (CS) in combination with glucosamine (GlcN) sulfate have been increasingly employed in therapies for osteoarthritis and osteoarthrosis [3]. The pharmaceutical preparations of CS present in this combination drug are obtained from cartilages of different animals and thus may present structural differences [4], especially regarding to variations in the 4- and 6-sulfation of *N*-acetyl β-d-galactosamine residues and minor differences in the 2-sulfation of glucuronic acid units (Figure 1A). CS from different animal sources may also present significant differences in their molecular weight [5].

In addition to the structural heterogeneity of CS obtained from different animal sources, some pharmaceutical preparations also present contaminations with other GAGs due to flaws during the purification process [5,6,7]. A common contaminant is keratan sulfate (KS), a conspicuous component of proteoglycans from cartilages [5,6,7]. Even the international standards of CS differ in their structures and purities. The CS standard from the United States Pharmacopeia purified from bovine cartilage has galactosamine units predominantly 4-sulfated (CS-A) while the European Pharmacopeia adopts CS obtained from shark cartilage, rich in 6-sulfated galactosamine (CS-C) and contaminated with minor amounts of KS [8,9]. The other pharmacologically active ingredient of these formulations is GlcN sulfate, which alone or in combination with CS ameliorates some symptoms of osteoarthritis [10,11,12]. The GlcN added in the formulations undergoes to mutarotation equilibrium between α- and β-anomers (~62% and ~38%, respectively) in solution (Figure 1B).

Different from heparin, which has a well-known anticoagulant and antithrombotic mechanism of action, the molecular basis of the therapeutic effects of CS and GlcN sulfate in the treatment of osteoarthritis and osteoarthrosis has not been fully determined [13]. Studies performed with chondrocytes undergoing osteoarthritis have been suggesting that CS might act targeting intermediates involved on cell signaling, inflammatory and catabolic pathways and on the oxidative stress [14,15,16,17]. Despite that uncertain mechanism of action, several preclinical tests, clinical trials and meta-analysis studies have clearly demonstrated the efficacy of CS combined with GlcN for the management of serious osteoarthritis symptoms such as pain, inflammation and cartilage degradation [18,19,20,21,22,23,24,25].

**Figure 2:**
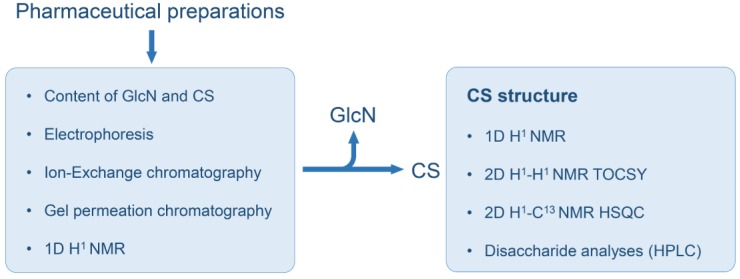
Systematic protocol for analyses of pharmaceutical preparations of CS-GlcN. The whole formulations were assessed via colorimetric reactions for determination of GlcN and CS contents, electrophoresis, chromatography and 1D ^1^H-NMR. Afterward, the CSs isolated from these formulations were further analyzed via 1D and 2D NMR and SAX-HPLC for determinations of the disaccharides formed after digestion with chondroitin AC lyase.

Since the preparations of CS prescribed for treatment of osteoarthritis and osteoarthrosis present extensive chemical variations depending on their animal source, it is necessary to establish a comprehensive set of analytical protocols to assess the fine structure, physicochemical characteristics and purity of each type of CS present in these pharmaceutical products. In the present study, we employed a systematic set of analytical methodologies to assess two different pharmaceutical products composed of GlcN in combination with CS purified from shark or bovine cartilages. Different batches of these formulations were initially assessed for their CS and GlcN content; afterward, the CS isolated from the batches was analyzed via anion-exchange and size-exclusion chromatography, agarose-gel electrophoresis and one- (1D) and two-dimensional (2D) nuclear magnetic resonance spectroscopy (NMR). We also compared preparations of CS from bovine cartilage in two different pharmaceutical forms (capsule and sachet) using 1D ^1^H NMR and analyses of the disaccharides formed after digestion with chondroitin AC lyase. The results of this set of analysis clearly demonstrate that the protocol proposed here (Figure 2) is robust enough for a clear and thorough characterization of pharmaceutical preparations of CS in combination with GlcN.

## 2. Materials and Methods

### 2.1. Pharmaceutical Formulations of CS Combined with GlcN and CS Standards

Sixty five batches of pharmaceutical grade preparations of CS (50 containing shark CS and 15 bovine CS) in combination with GlcN commercially available for oral administration as capsules or sachets were obtained from two different Brazilian pharmaceutical companies. The contents of both sachets and capsules were readily soluble in water. The names of the manufacturers were kept anonymous due to ethical principles. Standards of CS were obtained from the US (Rockville, MD, USA; cat. 1133570, Lot HOF184) and the European (Strasbourg, France; code Y0000593, ID. 002SJ4) pharmacopeias. Commercially available CS from whale cartilage (CS-A, mostly 4-sulfated) and shark cartilage (CS-C, mostly 6-sulfated) were purchased from Sigma-Aldrich (St. Louis, MO, USA). Standard of oversulfated CS (Lot No. HOM191) was obtained from US Pharmacopeia.

### 2.2. Chemical Analyses of the CS and GlcN Contents

The content of uronic acid in the CS from the pharmaceutical preparations was measured using carbazole reaction [26]. Briefly, solutions (200 μL) with different amounts of the preparations (10 → 80 μg) were incubated in 98% sulfuric acid and 0.9% sodium tetraborate (1.0 mL) at 100 °C for 12 min. The reaction was stopped in ice cold bath and then 0.2% carbazole in absolute ethanol (40 µL) was added to the solution and incubated at 100 °C for 5 min. The absorbance was measured at 525 nm in a spectrophotometer (Amershamn Biosciences, Little Chalfont, UK). The concentration of CS was estimated using a standard curve plotted with standard CS. The amount of GlcN in the pharmaceutical preparations was estimated via a colorimetric reaction [27]. Briefly, solutions (200 μL) with different amounts of the preparations (10 → 200 μg) were incubated in acetylacetone (5 mL Na_2_CO_3_ 1.25 N + 150 μL acetylacetone) at 100 °C for 20 min and cooled in ice cold. Then, Blix reagent (200 μL, 1.6 g *p*-dimetylaminobenzaldehide in 30 mL HCl + 30 mL ethanol 96%) were added to the solutions followed for another addition of 200 μL ethanol 96% and kept at room temperature for 1 h. The absorbance was measured at 530 nm in a spectrophotometer (Amersham Biosciences). The concentration of GlcN was estimated using a standard curve plotted with a solution of free GlcN.

### 2.3. Agarose-Gel Electrophoresis

Solutions containing CS from the pharmaceutical preparations and CS standards (5 μg of each) were applied in an agarose-gel (0.5%). The preparations submitted to enzymatic digestion were incubated with 0.01 units of chondroitin AC lyase from *Flavobacterium heparinum*, recombinant expressed in *E. coli* (Sigma-Aldrich) in 100 μL 0.05 M Tris:HCl (pH 8.0), supplemented with 5 mM EDTA and 15 mM sodium acetate at 37 °C for 12 h. After incubation, the samples were heated at dried-bath at 80 °C for 15 min to stop the reaction through denaturation of the enzyme, cooled in ice cold and applied in the agarose-gel. Electrophoresis run for 1 h at 110 V in 0.05 M 1,3-diaminopropane acetate (pH 9.0). CS in the gel were fixed with 0.1% *N*-cetyl-*N*,*N*,*N*-trimethylammonium bromide solution for 12 h, dried and stained with 0.1% toluidine blue in 0.1:5:5 (*v*/*v*) acetic acid:ethanol:water.

### 2.4. Anion-Exchange Chromatography

CS from the pharmaceutical formulations and CS standards (1 mg of each) were applied to a anion-exchange column Mono-Q (GE Healthcare, Little Chalfont, UK) pre-equilibrated with 10 mM Tris:HCl containing 0.5 M NaCl, pH 7.4 and connected to a HPLC system (Shimadzu, Kyoto, Japan). The column was eluted at a flow rate of 1.0 mL·min^−1^ using a linear NaCl gradient of 0.5 → 3.0 M NaCl, total volume of 40 mL. The eluent was continuously monitored via UV at 215 nm and conductivity.

### 2.5. Size-Exclusion Chromatography

The molecular weight of the formulations prepared with CS from shark or bovine cartilages and CS standards (20 μg of each) were evaluated via size-exclusion chromatography using a set of gel filtration columns (TSK gel G4000 SW × 1 and G3000 SW × 1, both 7.5 mm i.d. × 300 mm, Tosoh, Tokyo, Japan) linked to a HPLC system (Shimadzu). The columns were eluted with 0.1 M ammonium acetate, at room temperature with a flow rate of 0.3 mL·min^−1^. The eluent was monitored by refractive index. The columns were calibrated using a low-molecular-weight heparin molecular-weight standard (Lot No. 05/112) from the National Institutes of Biological Standards and Controls (NIBSC) and a heparin sodium molecular-weight calibrant from United States Pharmacopeia (Lot No. FOL4830), as previously described [2].

### 2.6. NMR Analyses

Spectra were obtained with CSs from shark or bovine cartilages present in the pharmaceutical preparations previously isolated using a syringe HITRAP desalting column (GE Healthcare). Spectra were recorded using a DRX 600 MHz apparatus (Bruker, Billerica, MA, USA) with a triple resonance probe as detailed previously [2]. About 20 mg of each sample was dissolved in 0.6 mL 99.9% deuterium oxide (Cambridge Isotope Laboratory, Cambridge, MA, USA). All spectra were recorded at 35 °C with HOD suppression by presaturation. The 1D ^1^H-NMR spectra were recorded using 16 scans and inter-scan delay set to 1 s. The 2D ^1^H–^1^H TOCSY spectra were recorded using states-time proportion phase incrementation (states-TPPI) for quadrature detection in the indirect dimension. The 2D ^1^H–^13^C edited-HSQC spectra was performed with 1024 × 256 points and globally optimized alternating phase rectangular pulses (GARP) for decoupling. Chemical shifts were displayed relative to external trimethylsilylpropionic acid at 0 ppm for ^1^H and relative to methanol for ^13^C.

### 2.7. Analysis of the Disaccharide Composition

The disaccharides were obtained through enzymatic digestion with chondroitin AC lyase as described in the Section 2.3. The disaccharide mixtures obtained from preparations of CS from bovine cartilage formulated as capsules and sachets and the CS standard were lyophilized and then dissolved in 100 µL of distilled water. The disaccharides (20 µL) were analyzed via strong anion-exchange chromatography on a 250 × 5 mm Spherisorb-SAX column (Sigma-Aldrich) linked to a HPLC system (Shimadzu). After the injection, the column was washed with 5 mL of acidified water (pH 3.5) and then the disaccharides were eluted from the column using a linear gradient of 0 → 1 M NaCl (pH 3.5) at a flow rate of 1 mL·min^−1^. The eluent was continuously monitored via UV at 232 nm.

## 3. Results and Discussion

Pharmaceutical preparations of bovine CS in combination with GlcN (CS-GlcN) for oral administration are available as capsule and sachet formulations. The precise amounts of each active compound present in the formulations was determined through specific colorimetric reactions for hexuronic acid and free GlcN contents. The CS and GlcN contents measured here were very close to those declared by the manufacturers for both formulations (Table 1). Preparations of shark CS-GlcN formulated as capsules presented proportions of the active compounds identical to those of bovine CS-GlcN capsules and in accordance with the declared by the manufacturers as well (data not shown). Both CS and GlcN present pharmacological activities related to the treatment of osteoarthritis symptoms [10,11,12]; therefore, a precise determination of the amounts of each one of these pharmacologically active ingredients is indispensable to confirm that each preparation is in accordance with the recommended posology.

**Figure 3:**
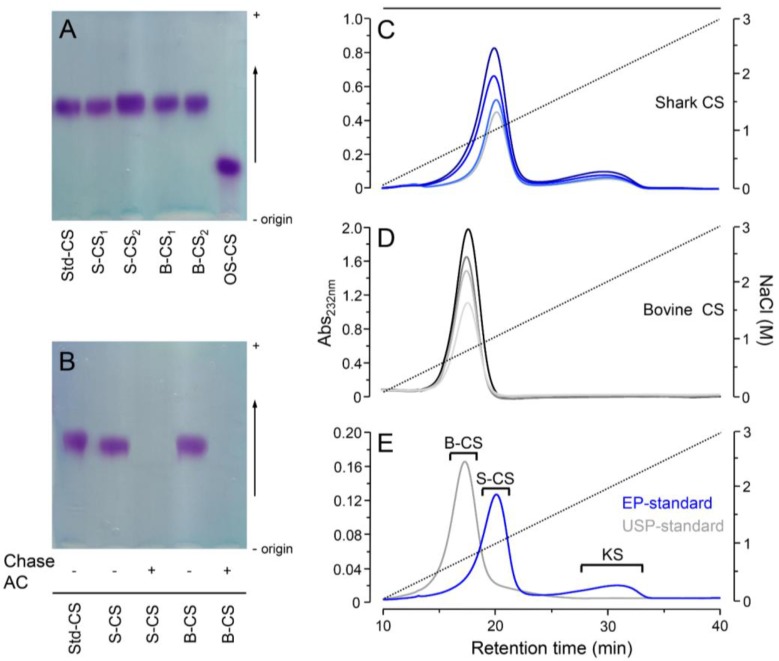
Agarose-gel electrophoresis and anion-exchange chromatography of pharmaceutical preparation of CS-GlcN. Agarose-gel electrophoresis of: (**A**) representative batches containing shark CS (S-CS in all panels) and bovine CS (B-CS in all panels) and standards of CS-A (Std-CS) and oversulfated CS (OS-CS) and (**B**) shark CS and bovine CS before (−) and after (+) digestion with chondroitin AC lyase. Anion-exchange chromatography of representative batches (50 → 200 µg) containing shark CS (panel (**C**), bluish lines), bovine CS (panel (**D**), greyish lines) and standards of CS (200 µg) from the European (in blue) and US (in grey) pharmacopeias (**E**), the peak containing keratan sulfate is also indicated (KS). Chromatography were performed using a Mono-Q column linked to a HPLC system through a linear gradient of 0.5 → 3.0 M NaCl (dotted lines). The eluents were monitored via UV (A_215nm_).

After the determination of the active compounds contents, we analyzed in further detail the CS from the different preparations. The first analytical steps toward determining the characteristics of the CS present in the different pharmacological preparations were analyses via agarose-gel electrophoresis and ion-exchange chromatography (Figure 3). The electrophoresis revealed that different batches of bovine and shark CS present electrophoretic mobility identical to the standard of CS (Figure 3A) and that both bovine and shark CS from the formulations are completely susceptible to digestion with chondroitin AC lyase (Figure 3B).

Despite this apparent homogeneity, we further investigated different batches containing shark or bovine CS using anion-exchange chromatography. The two compounds were clearly distinguishable in the chromatograms, with shark CS eluting at higher concentrations of NaCl than bovine CS (Figure 3C,D). Furthermore, the batches of shark CS presented small amounts (~16% of total) of another GAG, which was previously identified as keratan sulfate [5]. As expected, the international standards of CS from different animal sources also showed different chromatographic profiles. The standard of European Pharmacopeia, prepared from shark cartilage [8], elutes at higher concentration of NaCl and presents minor contamination with KS while the standard of US pharmacopeia purified from bovine cartilage [9] elutes at lower NaCl concentration and showed no contamination with other GAGs (Figure 3E). Therefore, the purity and homogeneity of the CSs from different animal sources present in the pharmaceutical preparations prescribed for treatment of osteoarthritis and osteoarthrosis are in strict accordance with the international standards.

Both the US and European pharmacopeias recommend cellulose-acetate electrophoresis stained with toluidine blue to assess the purity of pharmaceutical or nutraceutical preparations of CS [8,9]. This analysis is extremely important to detect the presence of other GAGs such as oversulfated chondroitin sulfate in the preparations. However, this technique has not enough resolution to differentiate neither CS with different chemical structure such as those from shark and bovine cartilage enriched with 6- or 4-sulfated galactosamine units, respectively, nor to detect minor contaminations with KS, even with samples previously digested with chondroitin AC lyase.

The inability of electrophoretic techniques to differentiate CS-A from CS-C and to detect small amounts of KS has already shown in analyses of pharmacological preparations of CS [5,28] and in studies on GAGs from other animal sources [29]. Otherwise, the anion-exchange chromatography analyses performed here and elsewhere [5,28] were plainly able to distinguish different CSs and to detect contaminations with KS. Therefore, we can affirm that anion-exchange chromatography is a robust analytical tool to perform evaluations on the source, homogeneity and purity of pharmaceutical preparations of CS.

**Figure 4:**
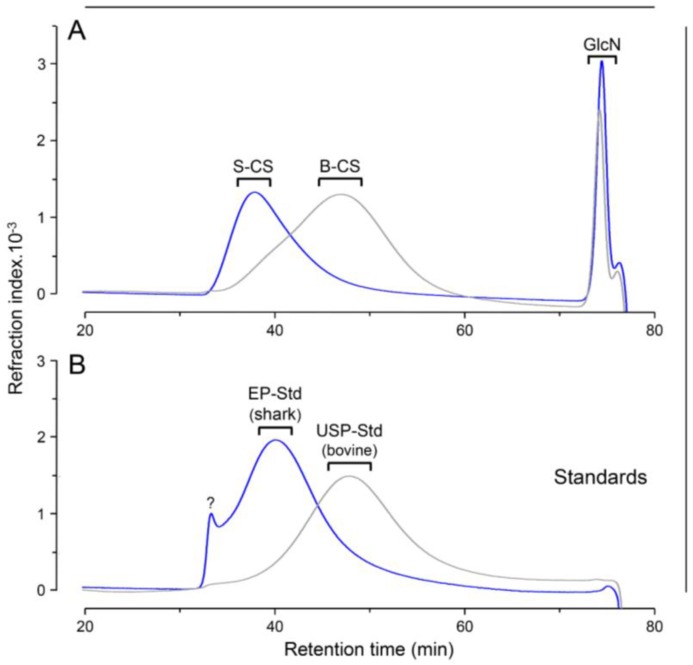
Size-exclusion chromatography of pharmaceutical preparation of CS-GlcN. (**A**) CS from preparations containing shark (S-CS) and bovine (B-CS) cartilages (blue and gray lines, respectively). (**B**) International standards from European and United State pharmacopeia (EP-Std in blue and USP-Std in gray, respectively). Chromatography were performed using a set of TSK gel G4000 SW/G3000 SW columns. The eluents were monitored via differential refractive index. GlcN in panel A indicates the elution of the glucosamine from the preparations and the ? in panel B depicts a non-identified component found in the standard from the European pharmacopeia.

We also compared the molecular weight of the different pharmaceutical preparations of CS-GlcN using a set of size-exclusion chromatography columns (Figure 4). Shark CS had markedly higher molecular weight than bovine CS (~50 KDa and ~23 KDa, respectively) and the GlcN of both preparations co-eluted as low-molecular weight components at the *V*_t_ of the columns (Figure 4A). As expected, the retention time of shark and bovine CS from the pharmaceutical preparations were similar to those of the standards of CS from European (shark) and US (bovine) pharmacopeias, respectively (Figure 4B). We also noticed a high molecular weight component in the standard of CS from the European Pharmacopeia eluting at the void volume of the columns (Figure 4B). This component probably consists of CS polymers with higher molecular weight, denoting a higher polydispersity of this standard in relation to the shark CS from the pharmaceutical preparation. Although the size-exclusion chromatography presents a good resolution to distinguish the different CSs, this technique has proven here and elsewhere [5] unable to detect KS contaminations in CS preparations from shark cartilage because the chromatograms yield a single and polydisperse peak.

**Figure 5:**
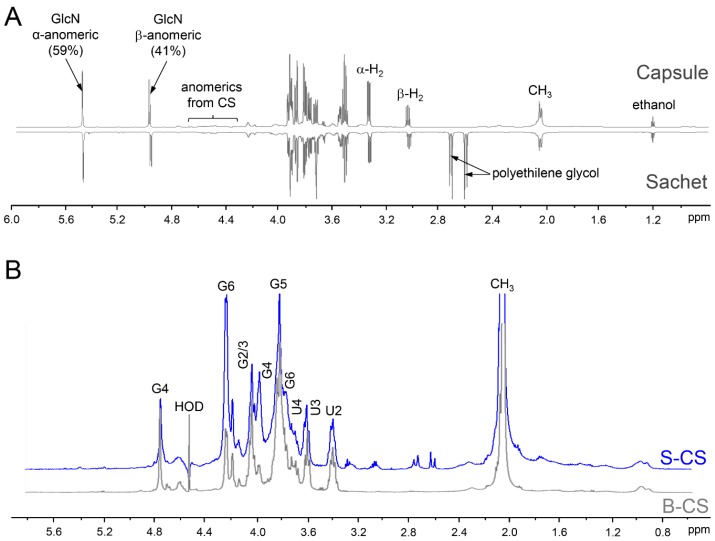
1D ^1^H NMR of the pharmaceutical preparation of CS-GlcN. (**A**) Wide range spectra of the preparations containing CS from bovine cartilage formulated as capsules and sachets; (**B**) Spectra of the CSs from shark (S-CS, in blue) and bovine (B-CS, in grey) cartilages isolated from the pharmaceutical preparations. The samples were dissolved on 0.4 mL of D_2_O and analyzed using a 600 MHz NMR spectrometer at 35 °C.

After the assessments on the purity and homogeneity of the different pharmaceutical preparations of CS-GlcN, we proceeded with the determination of the fine structures of their components using 1D and 2D NMR analysis. We first analyzed preparations of bovine CS-GlcN formulated as capsule or sachet via 1D ^1^H-NMR (Figure 5A). The spectra revealed two preponderant signals assigned to GlcN after mutarotation equilibrium. The proportions of α- (59%) and β-anomers (41%). were close to the expected values depicted in the panel B of Figure 1. Surprisingly, these α- and β-anomers had chemical shifts of their anomeric (α-H1 at 5.48 and β-H1 at 4.98 ppm) and ring protons coincident with those of non-sulfated instead of sulfated GlcN. No shifts indicating sulfation were noticed because the spectra were strictly coincident with that of standard non-sulfated GlcN (not shown). This finding could have pharmacological implications because the active compound present in these formulations (non-sulfated GlcN) differs from that approved for use by the regulatory agencies (viz. glucosamine sulfate sodium). Signals ascribed to CS are wide and discrete in the spectra, except for the clear CH_3_ signal from *N*-acetyl β-galactosamine at ~2.1 ppm. A signal of residual solvent (ethanol) was observed at 1.2 ppm. Formulations in capsule and sachet have the same active compounds, but present different excipients such as polyethylene glycol, which was easily identified by the multiplets at 2.6–2.8 ppm present in the spectrum of the sachet formulation. We were unable to compare the integrals of GlcN and CS in the spectra due to differences in relaxation properties of spins of sugars free in solutions and linked as polysaccharides.

To perform a precise analysis of the two types of CS present in the formulations we removed the GlcN and other excipients using a desalting column. The 1D ^1^H-NMR spectra of the purified CSs yielded well-defined signals (Figure 5B). CSs from shark and bovine cartilages (in blue and gray, respectively) showed the same set of signals but some of them present different intensities; notably, the signal at 4.74 ppm, ascribed to H4 of 4-sulfated *N*-acetyl β-galactosamine is more prominent in bovine than in shark CS. In contrast, signal from H6 of 6-sulfated *N*-acetyl β-galactosamine at 4.21 ppm is more intense in the shark CS. These observations indicate that the hexosamine residues of bovine CS are preponderantly 4-sulfated while those from shark CS are mostly 6-sulfated.

**Figure 6:**
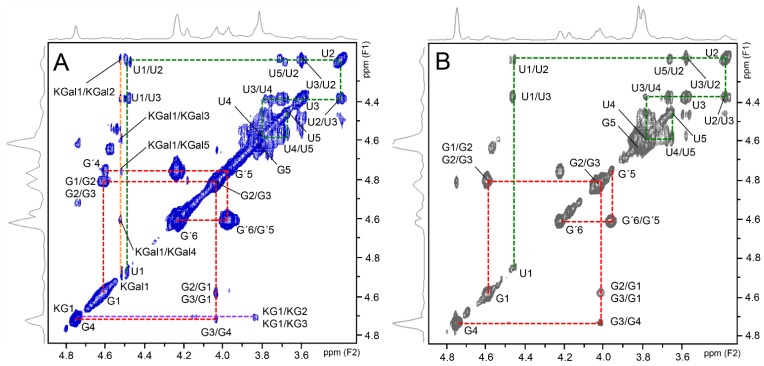
^1^H–^1^H TOCSY NMR spectrum of shark (**A**) and bovine (**B**) CS from the pharmaceutical preparations. Red and green lines in the panels represent the spin systems of *N*-acetyl β-galactosamine and of β-glucuronic acid of the CS, respectively. Note that the β-galactosamine have two distinct spin systems assigned to 4-sulfated residues (signals indicated as G) and 6-sulfated units (signals indicated as G′). Glucuronic acid has a single spin system (signals indicated as U). Shark CS has two additional spin systems assigned to 6-sulfated *N*-acetyl β-glucosamine (KG) and 6-sulfated β-galactose (KGal) units of keratan sulfate, represented by the purple and yellow lines, respectively.

Shark and bovine CSs also showed similar 2D ^1^H–^1^H TOCSY spectra with two preponderant spin systems (Figure 6), one assigned to 4- and 6-sulfated *N*-acetyl β-galactosamine units (signals indicated as G and G′, respectively, and connected by red lines) and the other assigned to β-glucuronic acid residues (signals indicated as U, connected by green lines). As expected, the spectrum of shark CS showed additional signals assigned to KS (Figure 6A). The spin system of the constituent units of KS, 6-sulfated *N*-acetyl β-glucosamine (assigned as KG) and 6-sulfated β-galactose (assigned as KGal) are connected by purple and yellow lines, respectively, in the spectrum (Figure 6A). The values of ^1^H chemical shifts of the constituent units of these GAGs are shown in Table 2.

**Figure 7:**
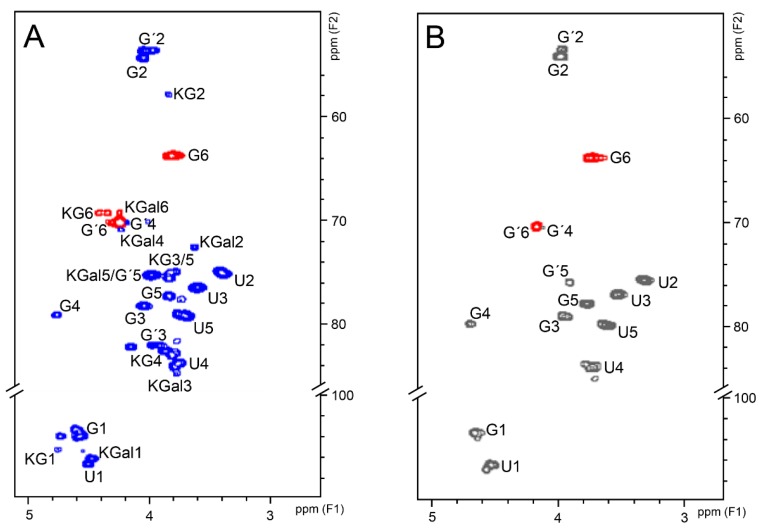
^1^H–^13^C HSQC spectra of shark (**A**) and bovine (**B**) CS from the pharmaceutical preparations. Blue (panel **A**) and gray (panel **B**) signals are in-phase and belong to CH groups whereas red signals are in antiphase and belong to CH_2_ groups. Signals assigned to 4-sulfated and 6-sulfated *N*-acetyl β-galactosamine are indicated as G and G′, respectively. Signals from β-glucuronic acid are indicated as U. Signals from *N*-acetyl β-glucosamine and β-galactose of keratan sulfate are indicated as KG and KGal, respectively.

2D ^1^H–^13^C HSQC spectra of the two types of CSs yielded well-resolved and similar signals (Figure 7). The spectrum of bovine CS is more homogenous (Figure 7B) while the spectrum of shark CS also shows some additional but less intense signals assigned to KS (Figure 7A) The signals attributed to KS (KG and KGal in the Figure 7A) were assigned by comparison with literature data [30]. The values of ^13^C chemical shifts of CS and KS present in the pharmaceutical preparations are shown in the Table 2. The integrals of H4/C4 and H6/C6 from 4-sulfated and 6-sulfated *N*-acetyl β-galactosamine, at 4.74/79.2 and 4.21/70.2 ppm, respectively, showed that shark CS is mostly 6-sulfated while bovine CS is preponderantly 4-sulfated (Table 3) and thus in strict accordance with the structure expected for these compounds [5].

These results have consistently demonstrated that 1D ^1^H and 2D ^1^H–^1^H TOCSY and ^1^H–^13^C HSQC NMR spectra of the preparations of GlcN-CS from different animal sources are informative enough to assess qualitatively and quantitatively the structures of the pharmacologically active ingredients (CS and GlcN) and excipients (polyethylene glycol and ethanol) present in the different formulations. Structural analyses via 1D and 2D NMR have been widely used to determine the chemical differences between porcine and bovine heparins and are routinely employed in the quality control of pharmaceutical grade unfractionated and low-molecular weight heparins [2,31]. Therefore, solution NMR can also be considered an effective analytical tool for structural determinations of the active ingredients of pharmaceutical formulations of GlcN-CS.

**Figure 8:**
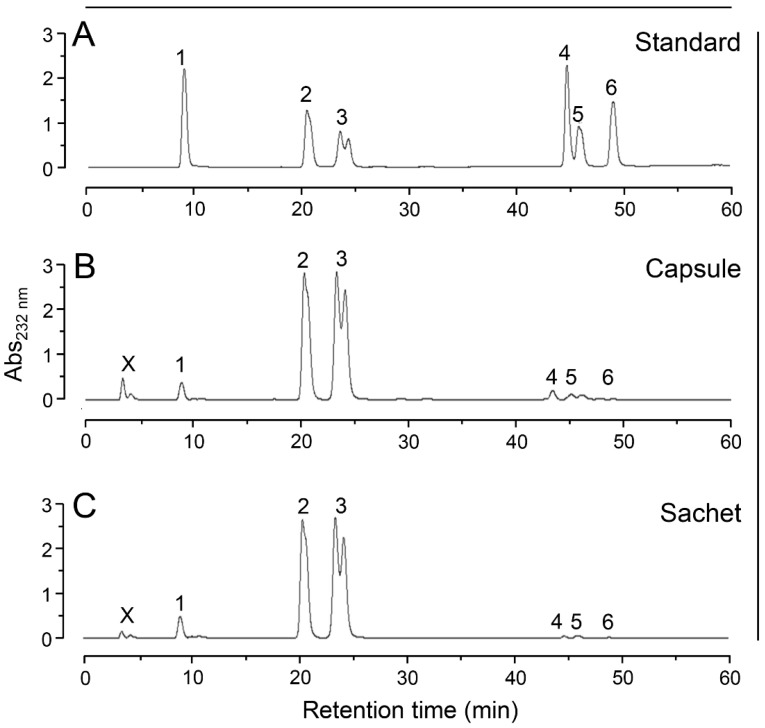
SAX-HPLC analyses of the disaccharides of different formulations containing bovine CS. Disaccharides were prepared through digestion with chondroitin AC lyase of standard of CS-A (**A**) and the bovine CS from capsule (**B**) and sachet (**C**) formulations. The mixtures of disaccharides were applied in a Spherisorb-SAX column linked to an HPLC system and eluted with a gradient of 0 → 1 M NaCl. The eluent was monitored for UV absorbance at 232 nm. The numbered peaks correspond to the elution positions of known disaccharide standards as follows: 1, ΔUA → GalNAc; 2, ΔUA → GalNAc (6SO_4_); 3, ΔUA → GalNAc (4SO_4_); 4, ΔUA (2SO_4_) → GalNAc (6SO_4_); 5, ΔUA → GalNAc (4,6-diSO_4_); 6, ΔUA(2SO_4_) → GalNAc (4SO_4_). X indicates a non-identified product. ΔUA → GalNAc (4SO_4_) shows two overlapped peaks assigned to the α- and β-anomers.

Additionally, we analyzed the disaccharide composition of the bovine CS present in the capsule or sachet formulation via SAX-HPLC chromatography (Figure 8). The disaccharides were prepared through enzymatic digestion of the CS isolated from the formulations with chondroitin AC lyase. We observed in both capsule and sachet formulations a preponderance of disaccharides containing 4-sulfate *N*-acetyl β-galactosamine, followed by units sulfated at position 6 and minor amounts of non-sulfated disaccharides. Only trace amounts di-sulfated disaccharides were detected in the chromatograms. CS from both capsule and sachet formulations presented chromatographic profiles similar to the CS-A standard (Figure 8A) and the quantification of their disaccharides are in accordance with literature data [32] (Table 4). We also include in the Table 4 the disaccharide composition of shark CS available in the literature [33], which were consistent with the NMR data obtained here.

## 4. Conclusions

Herein, we have performed a systematic assessment on the compositions, physicochemical characteristics and chemical structures of pharmaceutical formulations of CS in combination with GlcN prescribed to treat the symptoms of osteoarthritis and osteoarthrosis. Anion-exchange chromatography was proven more effective than agarose-gel electrophoresis to determine the type of CS present in the formulations (CS-A from bovine or CS-C from shark cartilages) as well as to detect contaminations with KS. However, electrophoretic analyses still relevant because they are able to readily detect contaminations with other GAGs, specially, oversulfated CS, a contaminant found in pharmaceutical preparations of heparin that provoked serious adverse effects and some deaths in the late 2000 years [34]. As demonstrated previously [5], size-exclusion chromatography is also unable to detect KS contaminations and thus ineffective to evaluate the purity of these pharmacological preparations.

Structural analysis via 1D ^1^H-NMR of the pharmacologically active ingredients revealed the presence of non-sulfated instead of sulfated GlcN in both capsule and sachet formulations. This finding is worrying because all the preclinical and clinical tests on the safety and efficacy of GlcN aiming the treatment of osteoarthritis were carried on with the sulfated compound [10,11,12]. The US pharmacopeia monograph recommends infrared absorption and dosage of sulfate content to assess the chemical characteristics of GlcN [35]; however, these techniques are not precise and/or specific enough to determine the presence and proportions of sulfated and non-sulfated GlcN in the pharmaceutical preparations. In contrast, the 1D ^1^H NMR analysis performed here was able to determine precisely whether the GlcN is sulfated or non-sulfated through the specific chemical shifts of their α- and β-anomers and, furthermore, it is potentially useful to quantify mixtures of these different sugars in pharmaceutical formulations by integrating their specific signals. Moreover, both 1D and 2D NMR spectra were useful for a precise determination of the chemical structure of the CS-A from bovine and CS-C from shark cartilages and the contaminant KS.

CS and GlcN alone or in combination have been massively marketed worldwide as over the counter dietary supplement or prescribed as a symptomatic slow-acting drug for osteoarthritis [11,36]. Both the US and European pharmacopeias monographs on these active compounds recommend only infrared spectroscopy and/or electrophoresis as analytical techniques to assess their purity and chemical compositions [8,9,35]. However, these techniques are not robust enough to determine: (1) the type of CS (CS-A or CS-C) present in the formulations; (2) the presence and quantity of contaminant KS and (3) the chemical composition of the GlcN (proportion of sulfated and non-sulfated sugars) present in the combinations. A recent update in the US pharmacopeia monograph on CS includes the analysis of disaccharide composition after digestion with chondroitim AC lyase [8]. In this update was recommended a ratio of 4-sulfated/6-sulfated disaccharides above one, this requirement cannot be fulfilled by preparations containing CS from shark cartilage, rich in 6-sulfated *N*-acetyl galactosamine units. Therefore, even this recently improved monograph is not comprehensive enough to evaluate the different types of CS adopted by the manufacturers.

Taking into consideration that the pharmaceutical preparations of CS-GlcN analyzed here presented extensive variations in their compositions such as different CS types, contamination with another GAG and GlcN with incorrect composition, more comprehensive and in-depth guidelines contemplating different CS types and inconsistent GlcN composition must be urgently drafted by the regulatory agencies. Besides the analytical techniques currently recommended by the pharmacopeias, we further suggest anion-exchange chromatography to evaluate the homogeneity and purity of the CS present in the pharmaceutical formulations. We also recommend the use of 1D ^1^H NMR instead of infrared spectroscopy to assess the chemical structure of both CS and GlcN present in this combination drug. The full implementation of the set of analytical protocols proposed here certainly will yields pharmaceutical preparations of CS-GlcN with more precise composition and hence with improved quality.

The mechanisms of action of neither CS nor GlcN in cartilage and subchondral bone tissues affected with osteoarthritis still not fully determined [14,15,16,17]. However, several in vivo studies with experimental animals and clinical trials already showed the effectiveness of these GAG-based compounds to ameliorate serious symptoms of osteoarthritis such as pain and cartilage degradation [18,19,20,21,22,23,24,25]. Nevertheless, it is a challenging task to understand how a carbohydrate-based compound with high molecular weight like CS (20–50 kDa) could be absorbed after oral administration and then remain sufficiently undegraded, even with the action of bacteria and enzymes of the gastrointestinal tract, to exert its therapeutic effects in the cartilages and bones. This lack of information reinforces the necessity of a precise and detailed determination of the chemical structures of the CS and GlcN present in these pharmaceutical preparations to support clinical and preclinical studies on their route of administration and mechanism of action.

## Figures and Tables

**Table 1:** Declared and observed contents of glucosamine (GlcN) and chondroitin sulfate (CS) on pharmaceutical preparations.

Formulation	Component	Declared Content (mg)	Observed Content (mg) ^a^
Capsule	GlcN ^b^	500	465 ± 20 ^b^
	CS ^c^	400	376 ± 8 ^c^
Sachet	GlcN	1500	1500 ± 60 ^b^
	CS	1200	1211 ± 179 ^c^

^a^ Results as mean ± SD of three determinations; ^b^ Content of free GlcN determined by a colorimetric reaction [27]; ^c^ Content of CS determined by the carbazole reaction [26] using standard curves obtained with the international standard of CS from US Pharmacopeia.

**Table 2:** ^1^H- and ^13^C-NMR chemical shifts of the constituent units of chondroitin sulfate (CS) and keratan sulfate (KS) found in the pharmaceutical preparations.

Signal	Chemical Shifts (ppm)				
	CS			KS	
	GalNAc-4S	GalNAc-6S	GlcA	GalNAc-6S	Gal-6S
**H1/C1**	4.58/103.6	4.58/103.6	4.46/106.5	4.74/105.2	4.56/105.3
**H2/C2**	4.02/54.0	4.02/53.4	3.37/75.2	3.85/57.6	3.62/72.3
**H3/C3**	*4.00/78.5* ^a^	*3.98/81.7*	3.57/76.2	3.83/75.3	*3.78/84.6*
**H4/C4**	**4.76/79.2 ^a^**	4.20/70.3	*3.77/83.8*	*3.87/82.5*	4.22/70.6
**H5/C5**	3.83/77.4	3.97/75.1	3.66/79.5	3.81/74.7	3.97/75.0
**H6/C6**	3.77/63.8	**4.24/70.1**	-	**4.41–4.33/69.0**	**4.24/69.0**

^a^ Sulfation and glycosylation sites are indicated in bold and italic, respectively.

**Table 3:** Proportions of 4- and 6-sulfated *N*-acetil β-galactosamine in bovine and shark chondroitin sulfates (CS) based on signals of 2D ^1^H–^13^C HSQC spectra (see Figure 5).

	Bovine CS	Shark CS
**4-sulfated β-GalNAc ^a^**	68%	16%
**6-sulfated β-GalNAc ^b^**	32%	84%

^a^ Signal at 4.74/79.2 ppm in the 2D ^1^H–^13^C HSQC spectra (see Figure 5); ^b^ Signal at 4.21/70.2 ppm in the 2D ^1^H–^13^C HSQC spectra (see Figure 5).

**Table 4:** Proportions of the disaccharides (% of total) produced through cleavage of chondroitin sulfate (CS) with chondroitin AC lyase.

Dissacharides	Bovine CS			Shark CS
	Capsule	Sachet	Literature Data ^a^	Literature Data ^b^
**ΔUA-GalNAc**	3	2	2	2
**ΔUA-GalNAc-6SO_4_**	36	36	41	76
**ΔUA-GalNAc-4SO_4_**	59	59	67	15
**ΔUA-2SO_4_-GalNAc-6SO_4_**	1	1	<1	7
**ΔUA-GalNAc-4,6diSO_4_**	1	2	<1	<1
**ΔUA-2SO_4_-GalNAc-6SO_4_**	<1	<1	<1	<1
**4-sulfation of β-GalNAc**	60	58	49	15
**6-sulfation of β-GalNAc**	38	39	35	83
**2-sulfation of β-GlcA**	1	1	<1	7

^a^ Data derived from Lauder et al. [32]; ^b^ Data from de Waard et al. [33].
